# Electrospun PLA/chitosan/PEG nanofibers for controlled delivery of ethyl *p*-methoxycinnamate

**DOI:** 10.3389/fbioe.2026.1831144

**Published:** 2026-06-24

**Authors:** Thi Dinh Do, Minh Ha Le, Thi Thuy Luyen Bui

**Affiliations:** 1 Hai Duong Central College of Pharmacy, Hai Duong, Vietnam; 2 Faculty of Pharmaceutical Chemistry and Technology, Hanoi University of Pharmacy, Hanoi, Vietnam; 3 Institute of Natural Products Chemistry, Vietnam Academy of Science and Technology, Hanoi, Vietnam

**Keywords:** biodegradable polymer membranes, controlled drug release, electrospinning, ethyl p-methoxycinnamate, PLA/chitosan nanofibers

## Abstract

**Background:**

Electrospun nanofibers have attracted considerable attention as drug delivery platforms due to their high surface area, interconnected porosity, and tunable structure. Ethyl p-methoxycinnamate (EPMC), a bioactive cinnamate derivative from Kaempferia galanga, exhibits anti-inflammatory and antioxidant activities, but its direct application may be limited by poor stability and uncontrolled release behavior. Incorporating EPMC into biodegradable electrospun polymer systems may improve its delivery performance.

**Methods:**

In this study, EPMC-loaded poly(lactic acid)/chitosan/polyethylene glycol (PLA/CS/PEG) nanofiber membranes were fabricated by electrospinning. Formulation composition and processing parameters were systematically evaluated. The resulting nanofibers were characterized using scanning electron microscopy, FTIR spectroscopy, contact angle measurement, and mechanical testing. Drug loading efficiency and *in vitro* release behavior were evaluated using UV–Vis spectroscopy, and release kinetics were analyzed using zero-order, first-order, Higuchi, and Korsmeyer–Peppas models.

**Results:**

Stable electrospinning conditions were obtained at 10 wt% PLA, 0.5 wt% chitosan, 1.0 wt% PEG, 20 kV applied voltage, 15 cm tip-to-collector distance, and a feed rate of 1.0 mL h^-1^. Drug incorporation resulted in a gradual increase in fiber diameter and markedly improved membrane wettability, while slightly reducing mechanical strength. Encapsulation efficiency decreased with increasing EPMC loading. The nanofiber membranes exhibited sustained drug release over 24 h, reaching approximately 51% cumulative release for the 15 wt% formulation. Kinetic analysis indicated that EPMC release followed the Korsmeyer–Peppas model, suggesting a combined diffusion–relaxation mechanism.

**Conclusion:**

These findings demonstrate that PLA/CS/PEG electrospun nanofibers provide an effective biodegradable carrier for controlled delivery of EPMC. The selected PLA/CS/PEG nanofiber membrane represents a promising biodegradable platform for controlled delivery of bioactive natural compounds such as EPMC.

## Introduction

Electrospinning has emerged as one of the most versatile and effective techniques for producing nanofibrous materials with diameters ranging from several micrometers down to tens of nanometers. Owing to their high specific surface area, interconnected porous structure, and tunable morphology, electrospun nanofibers have attracted considerable attention for biomedical applications, including tissue engineering scaffolds, wound dressings, and drug delivery systems (Liu et al., 2023; Mayilswamy et al., 2023). In particular, electrospun membranes provide unique advantages for controlled drug release due to their large surface-to-volume ratio and adjustable fiber architecture, which allow precise modulation of diffusion pathways and polymer–drug interactions (Forouzande et al., 2025; Kan et al., 2022). As a result, electrospinning has become a widely used strategy for developing advanced functional biomaterials and controlled drug delivery platforms (Li and Xia, 2004; Greiner and Wendorff, 2007; Liu et al., 2022).

Among the polymers commonly used in electrospinning, poly(lactic acid) (PLA) is one of the most extensively investigated biodegradable polymers because of its biocompatibility, mechanical strength, and renewable origin. PLA-based nanofibers have been widely explored in biomedical fields such as tissue scaffolds and drug delivery matrices (Sulutas et al., 2024). However, the hydrophobic nature of PLA may limit its interaction with biological environments and affect drug release performance. To overcome these limitations, blending PLA with hydrophilic polymers has been proposed as an effective strategy to improve the physicochemical properties of electrospun materials. Chitosan, a naturally derived polysaccharide obtained from chitin, possesses excellent biocompatibility, biodegradability, antimicrobial activity, and the ability to interact with a variety of bioactive molecules (Samokhin et al., 2023; Dash et al., 2011). The combination of PLA with chitosan has been shown to enhance hydrophilicity, biological activity, and drug loading capacity of electrospun nanofibers. In addition, polyethylene glycol (PEG) is frequently incorporated as a modifying polymer to further improve flexibility, wettability, and drug diffusion characteristics of composite electrospun membranes (Samokhin et al., 2023).

Ethyl *p*-methoxycinnamate (EPMC) is a naturally occurring cinnamate derivative that has been widely reported as a major bioactive compound in *Kaempferia galanga* L. (Zingiberaceae) (Wang et al., 2023; Munda et al., 2018). This compound has attracted attention due to its diverse biological activities, including anti-inflammatory, antioxidant, antimicrobial, and UV-absorbing properties (Umar et al., 2014). Because of these properties, EPMC has potential applications in pharmaceutical and biomedical products such as topical formulations, skin protection systems, and functional biomaterials. However, the direct use of free EPMC may be limited by its stability and uncontrolled release behavior. Incorporating EPMC into polymer-based delivery systems is therefore considered a promising approach to improve its stability, control release kinetics, and enhance its biomedical applicability (Tiong et al., 2026).

Despite the growing interest in electrospun nanofiber-based drug delivery systems, most studies have primarily focused on conventional synthetic drugs or widely studied natural compounds (Tiong et al., 2026; Bhardwaj and Kundu, 2010). In contrast, the incorporation of ethyl p-methoxycinnamate (EPMC), a bioactive cinnamate derivative with notable anti-inflammatory and antioxidant properties, into electrospun biodegradable polymer matrices has received limited scientific attention. In particular, the development of composite nanofiber systems based on poly(lactic acid) (PLA) and chitosan for the controlled delivery of EPMC remains largely unexplored. Although PLA provides good mechanical strength and processability for electrospinning, its hydrophobic nature may restrict drug diffusion and interaction with biological environments (Samokhin et al., 2023; Auras et al., 2004). Meanwhile, chitosan and polyethylene glycol (PEG) have been shown to enhance hydrophilicity, bioactivity, and drug release behavior in polymeric systems (Samokhin et al., 2023; Dash et al., 2011; Mouro et al., 2023), but their combined effects on EPMC-loaded electrospun nanofibers have not been systematically investigated.

Furthermore, the relationships between formulation composition, electrospinning parameters, fiber morphology, and drug release kinetics in such composite systems remain insufficiently understood. A comprehensive evaluation of these factors is essential for designing nanofibrous materials capable of achieving stable structure, efficient drug loading, and controlled release behavior suitable for biomedical applications. Therefore, the present study aimed to fabricate EPMC-loaded PLA/chitosan/PEG nanofiber membranes using electrospinning and to systematically investigate the influence of formulation composition and electrospinning parameters on fiber morphology, surface wettability, drug loading efficiency, and *in vitro* release behavior.

## Materials and Methods

### Materials

Polylactic acid (PLA) pellets (diameter 1.75 mm) were used as the primary biodegradable polymer matrix. The PLA used in this study had an average molecular weight of approximately 100,000 g mol^-1^ and was supplied by NatureWorks LLC (USA). Chitosan (CS) with a degree of deacetylation ≥90% and a medium molecular weight range of approximately 100–300 kDa was used as a hydrophilic biopolymer to enhance the biological compatibility and wettability of the electrospun fibers. Chitosan was obtained from Sigma–Aldrich (St. Louis, MO, USA). Polyethylene glycol (PEG 4000) was employed as a modifying polymer to improve flexibility and regulate drug release behavior within the composite matrix. PEG 4000 (average molecular weight 4000 g mol^-1^) was purchased from Merck (Darmstadt, Germany).

Ethyl p-methoxycinnamate (EPMC), a naturally occurring cinnamate derivative, was used as the model bioactive compound incorporated into the nanofiber system. EPMC with a purity of ≥98% was obtained from Tokyo Chemical Industry (TCI, Tokyo, Japan).

Chloroform (≥99.0%) was used as the solvent for dissolving PLA, while acetic acid (≥99.5%) was used for preparing chitosan solutions. Analytical-grade chloroform and glacial acetic acid were purchased from Merck (Darmstadt, Germany) and used as received. Phosphate-buffered saline (PBS, pH 7.4) was used as the release medium in drug release studies. PBS tablets were obtained from Sigma–Aldrich and dissolved in deionized water to prepare the buffer solution.All chemicals were of analytical grade and used without further purification. Deionized water with a resistivity of 18.2 MΩ cm was used in all aqueous preparations.

Electrospinning was performed using a laboratory-scale electrospinning apparatus equipped with a high-voltage power supply (0–30 kV), a programmable syringe pump, and a rotating drum collector. Morphological analysis of the electrospun fibers was carried out using scanning electron microscopy (SEM). Surface wettability was evaluated using a contact angle analyzer. UV–Vis spectroscopy was employed for quantification of EPMC concentration in release studies, while fiber diameter distribution was analyzed using ImageJ software (National Institutes of Health, USA).

### Preparation of electrospinning solutions

Polymer solutions for electrospinning were prepared in several steps to ensure homogeneous mixing and adequate viscosity for fiber formation. All solutions were prepared using analytical-grade reagents under controlled laboratory conditions.

First, PLA was dissolved in chloroform at predetermined concentrations. In the present study, PLA concentrations ranging from 8% to 12% (w/v) were investigated to identify suitable solution viscosity for electrospinning. The polymer was weighed accurately and placed in a sealed glass vessel containing chloroform. The mixture was heated to approximately 70 °C and stirred magnetically at 300 rpm until a clear and homogeneous solution was obtained. Typically, complete dissolution was achieved after approximately 2–3 h of continuous stirring.

Separately, chitosan solutions were prepared by dissolving CS in aqueous acetic acid under magnetic stirring at 60 °C. Chitosan was dissolved in 1% (v/v) aqueous acetic acid to obtain solutions with concentrations ranging from 0.25% to 0.75% (w/v). The temperature was gradually increased to approximately 80 °C to facilitate complete dissolution and formation of a uniform solution. The solution was stirred until no visible particles remained, indicating complete dissolution.

The CS solution was then slowly added to the PLA solution under continuous stirring to obtain a PLA/CS polymer blend. The addition was performed dropwise to minimize phase separation between the organic PLA solution and the aqueous chitosan phase. PEG was subsequently introduced into the mixture as a hydrophilic modifier. PEG 4000 was added at concentrations between 0.5% and 1.5% (w/v) relative to the total polymer content. The resulting PLA/CS/PEG solution was stirred at reduced speed (100 rpm) for approximately 24 h to ensure complete homogenization of the polymer blend. After stirring, the solution was allowed to stand at room temperature for several hours to facilitate the removal of entrapped air bubbles prior to electrospinning.

For drug-loaded systems, EPMC was added to the polymer solution at predetermined concentrations and allowed to dissolve under continuous stirring for 24 h prior to electrospinning. EPMC was incorporated at loading levels of 10–20 wt% relative to the total polymer mass. The drug-containing solutions were protected from light and stirred at ambient temperature to ensure complete dissolution and uniform dispersion of the bioactive compound within the polymer matrix before electrospinning.

### Fabrication of nanofibers by electrospinning

Electrospun nanofiber membranes were fabricated using a syringe-based electrospinning system. The electrospinning setup consisted of a high-voltage power supply (0–30 kV), a programmable syringe pump, and a grounded rotating drum collector covered with aluminum foil. The prepared polymer solution was loaded into a 5 mL syringe fitted with a stainless steel needle (G21, inner diameter approximately 0.5 mm). The syringe was mounted on a syringe pump to control the feed rate during electrospinning.

Electrospinning experiments were performed under ambient laboratory conditions at a temperature of approximately 25 °C ± 2 °C and a relative humidity of 50% ± 5%. During the electrospinning process, a high-voltage power supply was applied between the needle tip and the grounded collector. Under the applied electric field, a polymer jet was ejected from the needle tip and stretched toward the collector, forming continuous nanofibers as the solvent evaporated in flight. The resulting fibers were deposited onto the rotating drum collector. The collector rotation speed was maintained at approximately 200 rpm to facilitate uniform deposition of the nanofiber membranes.

Electrospinning parameters including applied voltage, tip-to-collector distance, and feed rate were systematically varied to determine suitable conditions for stable fiber formation. The applied voltage was varied between 10 and 24 kV, the tip-to-collector distance between 10 and 20 cm, and the solution feed rate between 0.5 and 1.5 mL h^-1^ during the systematic evaluation of electrospinning parameters. Each electrospinning condition was tested in at least three independent experiments to ensure reproducibility of fiber morphology.

After electrospinning, the resulting nanofiber membranes were carefully removed from the collector and transferred to a vacuum dryer at 40 °C for 24 h to remove residual solvent prior to further characterization.

### Experimental design

A systematic experimental design was employed to evaluate the influence of formulation composition and electrospinning parameters on fiber morphology and drug delivery properties. The experimental workflow consisted of sequential evaluation of polymer composition followed by evaluation of electrospinning parameters.

Initially, the concentration of PLA in chloroform was investigated at different levels to determine a suitable polymer concentration for stable electrospinning. PLA concentrations ranging from 8% to 12% (w/v) were examined to identify the range that could produce continuous fibers without bead formation.

Subsequently, the concentration of chitosan in the PLA matrix was varied to assess its effect on fiber formation and morphology. CS was incorporated at concentrations between 0.25% and 0.75% (w/v) relative to the solution to evaluate its influence on fiber diameter, uniformity, and surface characteristics. Although CS is generally insoluble in water, alkaline solutions, and most organic solvents, it readily dissolves in dilute acidic aqueous solutions due to protonation of its amino groups, which facilitates its incorporation into the electrospinning system.

After identifying suitable PLA and CS concentrations, PEG was incorporated as a hydrophilic modifier and its concentration was adjusted to investigate its influence on fiber structure and wettability. PEG 4000 was added at concentrations between 0.5% and 1.5% (w/v) relative to the total polymer content to assess its effect on fiber flexibility and surface hydrophilicity.

Finally, EPMC was introduced into the polymer system at different loading levels. Drug loading levels of 10–20 wt% relative to the total polymer mass were evaluated to investigate the influence of EPMC incorporation on fiber morphology, drug loading efficiency, and release behavior.

In addition to formulation variables, electrospinning parameters including applied voltage (10–24 kV), tip-to-collector distance (10–20 cm), and solution feed rate (0.5–1.5 mL h^-1^) were systematically investigated to determine their effects on fiber formation and uniformity. The suitable electrospinning conditions were selected based on stable jet formation, bead-free fiber morphology observed by SEM, narrow fiber diameter distribution, and adequate mechanical integrity of the resulting nanofiber membranes. All experiments were performed in at least triplicate to ensure reproducibility of the obtained results.

### Characterization of nanofibers

#### Morphology (SEM)

The morphology of the electrospun nanofiber membranes was examined using scanning electron microscopy (SEM). SEM observations were performed using a field-emission scanning electron microscope (FE-SEM, JSM-IT200, JEOL, Japan).Samples were mounted on aluminum stubs and coated with a thin conductive layer prior to imaging. A thin layer of gold was deposited on the samples using a sputter coater for approximately 60 s to improve surface conductivity. SEM micrographs were obtained at different magnifications to evaluate fiber structure, uniformity, and the presence of defects such as beads or droplets. Images were acquired at an accelerating voltage of 10–15 kV with magnifications ranging from ×1,000 to 100,00×.

#### Fiber diameter analysis

Fiber diameter distribution was determined from SEM images using ImageJ software. Image analysis was performed using ImageJ software (National Institutes of Health, USA). At least 100 individual fibers were randomly selected from each SEM micrograph to calculate average fiber diameter and standard deviation. For each sample, measurements were obtained from at least three independent SEM images to ensure statistical reliability. Frequency distribution histograms were generated to evaluate the uniformity of the fiber structure.

#### FTIR analysis

Fourier transform infrared spectroscopy (FTIR) was used to investigate chemical interactions between PLA, chitosan, PEG, and EPMC in the composite nanofibers. FTIR spectra were recorded using an FTIR spectrometer (Nicolet iS10, Thermo Scientific, USA) equipped with an attenuated total reflectance (ATR) accessory. Spectra were recorded within the range of 4000–400 cm^-1^ to identify characteristic absorption bands corresponding to functional groups of the polymer matrix and the incorporated bioactive compound. Each spectrum was collected with a spectral resolution of 4 cm^-1^ and averaged over 32 scans to improve signal-to-noise ratio.

#### Contact angle measurement

Surface wettability of the nanofiber membranes was evaluated by static water contact angle measurements. Contact angle measurements were performed using a contact angle goniometer (Dataphysics OCA 20, Germany). A droplet of distilled water was placed on the fiber surface and the contact angle was recorded using a contact angle analyzer. A water droplet with a volume of 3 μL was gently deposited on the membrane surface using a microsyringe. Measurements were performed at multiple positions on each sample and the average value was reported. For each membrane, at least five independent measurements were conducted at different locations and the results were expressed as mean ± standard deviation.

#### Mechanical testing

Mechanical properties of the nanofiber membranes were evaluated by tensile testing. Tensile tests were performed using a universal testing machine (Instron 3343, Instron Corporation, USA). Rectangular specimens were cut from the electrospun membranes and subjected to tensile loading until failure. Specimens were prepared with approximate dimensions of 10 mm × 30 mm and a gauge length of 20 mm. Parameters including tensile strength and elongation at break were recorded to assess the mechanical stability of the composite nanofiber system. The tests were conducted at a crosshead speed of 5 mm min^-1^ using a 100 N load cell. For each formulation, at least five specimens were tested and the average values with standard deviations were reported. The thickness of the electrospun membranes was measured using a digital micrometer (Mitutoyo, Japan) prior to tensile testing, and the average thickness from five measurements was used for stress calculation.

### Drug loading efficiency

Drug loading efficiency of EPMC in the electrospun membranes was determined by extracting the drug from a known amount of dried nanofiber membrane using an organic solvent capable of dissolving the polymer matrix. Approximately 10 mg of dried nanofiber membrane was accurately weighed and immersed in 10 mL of chloroform under gentle magnetic stirring to dissolve the polymer matrix and release the encapsulated EPMC. The resulting solution was then diluted with ethanol to obtain a suitable concentration range for spectroscopic analysis.

The solution was filtered through a 0.45 μm syringe filter to remove any insoluble residues prior to analysis. The concentration of EPMC in the solution was quantified using UV–Vis spectroscopy based on a calibration curve constructed at the maximum absorption wavelength of the compound. UV–Vis measurements were performed using a UV–Vis spectrophotometer (UV-1800, Shimadzu, Japan), and the absorbance was recorded at the characteristic wavelength of EPMC.

Drug loading efficiency was calculated by comparing the measured amount of EPMC with the theoretical amount initially incorporated into the polymer solution. Drug loading efficiency (DLE) was calculated according to the following equation:

DLE (%) = (Actual amount of EPMC extracted from the membrane/Theoretical amount of EPMC added during preparation) × 100.

All measurements were performed in triplicate, and the results were expressed as mean ± standard deviation.

### *In vitro* drug release

*In vitro* release behavior of EPMC from the nanofiber membranes was evaluated in PBS (pH 7.4) to simulate physiological conditions. Drug release experiments were conducted at 37 °C ± 0.5 °C using a thermostatically controlled orbital shaker. A known mass of drug-loaded nanofiber membrane (approximately 10 mg) was immersed in 20 mL of PBS (pH 7.4) under gentle agitation. The shaking speed was maintained at 100 rpm to ensure uniform mixing of the release medium.

At predetermined time intervals, 1 mL aliquots of the release medium were withdrawn and replaced with an equal volume of fresh PBS to maintain constant volume. The collected samples were filtered through a 0.45 μm syringe filter to remove any suspended fibers before analysis. The concentration of EPMC in the collected samples was measured using UV–Vis spectroscopy, using a UV–Vis spectrophotometer (UV-1800, Shimadzu, Japan) at the characteristic absorption wavelength of EPMC. A calibration curve was established prior to analysis to ensure accurate quantification.

Cumulative drug release was calculated based on the measured concentration and expressed as the percentage of EPMC released relative to the total amount of drug initially loaded in the membrane. All release experiments were performed in triplicate, and the results were reported as mean ± standard deviation.

### Release kinetic modeling

The drug release data obtained from the *in vitro* release study were fitted to several kinetic models to investigate the release mechanism of EPMC from the nanofiber matrix. These models included zero-order, first-order, Higuchi, and Korsmeyer–Peppas models. Curve fitting and regression analysis were performed using OriginPro software (OriginLab Corporation, USA).

The mathematical models used for the analysis are described below.

Zero-order model:

Qt=Q0+k0t

First-order model:

$$\ln\left(Q0\;-\text{ Qt}\right)=\ln Q0-k1t$$

Higuchi model:

Qt=kH t1/2

Korsmeyer–Peppas model:

$$\text{Mt }/\;M\infty =k\;t^{n}$$

where Qt is the amount of drug released at time t, Q0 is the initial amount of drug in the system, Mt/M∞ represents the fraction of drug released at time t, and k is the release rate constant corresponding to each model.

Among these models, the Korsmeyer–Peppas model was used to evaluate the release exponent (n), which provides insight into the dominant release mechanism. The value of n was obtained from the slope of the $\log\left(\text{Mt}/M\infty \right)$ *versus* log(t) plot.

Values of n between 0.5 and 1.0 indicate non-Fickian or anomalous transport behavior, suggesting that drug release is governed by a combination of diffusion and polymer relaxation processes. The goodness of fit for each kinetic model was evaluated using the correlation coefficient (*R* ^2^), and the model with the highest *R* ^2^ value was considered to best describe the release behavior.

### Statistical analysis

All experimental data were expressed as mean ± standard deviation (SD). Each experiment was performed with at least three independent replicates unless otherwise stated. Statistical analysis was performed using OriginPro software (OriginLab Corporation, USA). Differences between experimental groups were analyzed using one-way analysis of variance (ANOVA) followed by Tukey’s *post hoc* test for multiple comparisons. Prior to statistical testing, data distribution was examined to ensure the validity of parametric analysis. A p-value less than 0.05 (p < 0.05) was considered statistically significant.

### Replication and reproducibility

All formulations were prepared independently at least three times to ensure reproducibility of the electrospinning process.Characterization experiments including SEM imaging, fiber diameter measurements, contact angle analysis, mechanical testing, and drug release studies were performed in triplicate unless otherwise specified. The reported results represent the mean values obtained from independent measurements.

### Laboratory safety considerations

All experiments involving volatile organic solvents and electrospinning processes were conducted in accordance with standard laboratory safety protocols. Chloroform and acetic acid were handled in a well-ventilated chemical fume hood while wearing appropriate personal protective equipment (lab coat, gloves, and safety glasses). Electrospinning experiments were performed using a high-voltage power supply, and appropriate precautions were taken to ensure electrical safety during operation.

## Results and Discussion

### Evaluation of electrospinning parameters

The fabrication of uniform PLA/chitosan/PEG nanofiber membranes required a stepwise evaluation of formulation composition and electrospinning parameters. In the first stage, the effect of PLA concentration on fiber formation was examined. At 5 wt%, the solution viscosity was too low to support continuous jet elongation, and stable fiber formation was not achieved. Under this condition, the polymer chains were insufficiently entangled, leading to failure of jet stabilization and almost no fiber deposition. In contrast, when the PLA concentration was increased to 15 wt%, the solution became overly viscous, which hindered electrostatic stretching and promoted the formation of secondary jets and irregular thick segments. The fibers obtained at this concentration were much larger and less homogeneous. The intermediate concentration of 10 wt% provided the most favorable balance between viscosity and chain entanglement, enabling stable Taylor cone formation and the production of relatively smooth and uniform fibers, as summarized in Table 1.

**TABLE 1:** Effect of PLA concentration on fiber diameter and electrospinning behavior.

PLA concentration (wt.%)	Fiber formation	Mean diameter (µm)	Maximum diameter (µm)	Minimum diameter (µm)	Observation
5	No stable fiber	–	–	–	Insufficient viscosity, no continuous fibers
10	Stable	0.38 ± 0.08	0.57	0.23	Uniform fibers, smooth membrane
15	Unstable	1.19 ± 0.22	1.83	0.42	High viscosity, large fibers, secondary jets

This observation is consistent with the classical electrospinning theory, in which polymer chain entanglement and solution viscosity are critical parameters controlling jet stability and fiber formation (Casasola et al., 2016; Gupta et al., 2005). When the polymer concentration is too low, insufficient chain overlap results in the breakup of the jet into droplets, while excessively high viscosity restricts elongational flow and limits jet stretching under the electric field. Previous studies have shown that an optimal polymer concentration window is necessary to achieve continuous fiber formation and uniform morphology during electrospinning processes (Li and Xia, 2004; Huang et al., 2003).

Based on these findings, 10 wt% PLA was selected for subsequent experiments. After fixing PLA concentration, chitosan was added to improve hydrophilicity and biofunctionality of the polymer matrix. The incorporation of CS increased the viscosity of the solution and slightly enlarged the fiber diameter. At 0.5 wt% CS, the electrospinning process remained stable, the Taylor cone was maintained, and the resulting membranes were smooth and largely defect-free. Increasing CS to 1.0 wt% caused noticeable instability, including intermittent jet obstruction, irregular thinning, slack fiber segments, and bead-like defects. These changes likely resulted from excessive viscosity and increased resistance to electrostatic stretching. Therefore, 0.5 wt% CS was considered the most suitable concentration for the composite system, as shown in Table 2.

**TABLE 2:** Effect of chitosan concentration on fiber diameter and membrane quality.

CS concentration (wt.%)	Mean diameter (µm)	Maximum diameter (µm)	Minimum diameter (µm)	Membrane appearance	Interpretation
0.0	0.38 ± 0.08	0.57	0.23	Smooth	PLA control
0.5	0.43 ± 0.08	0.72	0.26	Smooth, uniform	Suitable viscosity and stable jet
1.0	0.52 ± 0.29	2.10	0.06	Defective, uneven	Excessive viscosity, unstable spinning

The increase in fiber diameter after adding chitosan can be attributed to the higher viscosity and intermolecular interactions introduced by the polysaccharide chains. Chitosan contains abundant hydroxyl and amino functional groups capable of forming hydrogen bonding networks within the polymer solution, which increases solution elasticity and reduces jet stretching during electrospinning. Similar effects have been reported in other PLA–chitosan electrospinning systems, where increasing CS concentration led to thicker fibers and reduced process stability due to increased solution viscosity and electrical resistance (Dash et al., 2011; Bhardwaj and Kundu, 2010; Poshina et al., 2019).

The applied voltage was then varied to refine the fiber morphology. At 10 kV, the electrostatic force was insufficient to fully stretch the PLA/CS jet, resulting in relatively large fibers. At 24 kV, the excessive electric field strength promoted the formation of secondary jets, which produced branched and uneven fibers and led to film defects. By comparison, 20 kV generated the most stable electrospinning behavior, maintaining a continuous Taylor cone and producing a smooth membrane without obvious beads or droplets. Although the mean fiber diameter at 24 kV was numerically slightly smaller, the morphology at this voltage was less homogeneous. Thus, 20 kV was retained as the suitable voltage, as presented in Table 3.

**TABLE 3:** Effect of applied voltage on PLA/CS fiber diameter.

Voltage (kV)	Mean diameter (µm)	Maximum diameter (µm)	Minimum diameter (µm)	Morphological assessment
10	0.43 ± 0.08	0.72	0.26	Incomplete stretching, larger fibers
20	0.38 ± 0.09	0.64	0.16	Uniform fibers, stable process
24	0.37 ± 0.07	0.58	0.14	Secondary jets, branching tendency

The influence of applied voltage on fiber morphology is related to the balance between electrostatic force and surface tension at the tip of the needle. Increasing the applied voltage enhances the electrical field strength and promotes jet stretching, which generally reduces fiber diameter. However, excessively high voltage can destabilize the jet and lead to the formation of secondary jets or branching phenomena, resulting in heterogeneous fiber morphology. Similar voltage-dependent behaviors have been widely reported in electrospinning studies of biodegradable polymer systems (Li and Xia, 2004; Greiner and Wendorff, 2007).

The tip-to-collector distance also affected the final fiber morphology. When the distance was too short (10 cm), solvent evaporation time was limited. When the distance was too long (20 cm), the jet became less stable and excessive stretching produced thin broken fibers in some regions. At 15 cm, the membrane surface was the most uniform, and the spinning process was steady. Interestingly, the average fiber diameter increased as the distance increased, which suggests that in this solvent system, prolonged jet travel may have promoted partial solvent loss and accumulation of thicker polymer segments before deposition. Therefore, a distance of 15 cm was selected, as was shown in Table 4.

**TABLE 4:** Effect of tip-to-collector distance on PLA/CS fiber diameter.

Distance (cm)	Mean diameter (µm)	Maximum diameter (µm)	Minimum diameter (µm)	Morphological assessment
10	0.38 ± 0.09	0.64	0.16	Acceptable, but less stable
15	0.46 ± 0.11	0.93	0.18	Uniform and defect-free membrane
20	0.49 ± 0.17	0.96	0.20	Broken fibers, reduced uniformity

The effect of the tip-to-collector distance can be explained by the combined influence of jet flight time and solvent evaporation dynamics. A shorter distance may lead to incomplete solvent evaporation and fiber fusion on the collector surface, whereas a longer distance may weaken the electric field intensity and reduce the stretching force acting on the jet. Consequently, an intermediate distance often provides the most stable electrospinning condition by balancing solvent evaporation and electrostatic elongation of the polymer jet (Huang et al., 2003).

The influence of feed rate was investigated under the selected PLA/CS conditions. A low feed rate of 0.5 mL h^-1^ caused inverted Taylor cone formation and nozzle clogging, while 1.5 mL h^-1^ produced polymer droplets and surface defects because the solution delivery exceeded the electrostatic drawing capacity. A feed rate of 1.0 mL h^-1^ offered the best balance between solution supply and jet stretching and was therefore used in subsequent experiments.

Feed rate plays a critical role in controlling the amount of polymer solution available at the needle tip during electrospinning. When the feed rate is too low, the polymer supply becomes insufficient to maintain a stable Taylor cone, which may cause jet interruption and needle blockage. Conversely, excessive feed rate can lead to incomplete stretching and the formation of beads or droplets due to the accumulation of solution at the needle tip. Therefore, a suitable feed rate must be selected to ensure a stable balance between solution supply and electrostatic stretching forces (Bhardwaj and Kundu, 2010).

PEG was then introduced to improve wettability and potential drug release behavior. Increasing PEG concentration from 1.0 to 2.0 wt% led to gradual enlargement of fiber diameter. At 2.0 wt%, secondary jets and local instabilities became visible, even though the average diameter increase was moderate. The 1.0 wt% PEG membrane showed the most suitable combination of structural uniformity and electrospinning stability and was selected as the most suitable composition for subsequent drug loading, as summarized in Table 5.

**TABLE 5:** Effect of PEG concentration on PLA/CS/PEG fiber diameter.

PEG concentration (wt.%)	Mean diameter (µm)	Maximum diameter (µm)	Minimum diameter (µm)	Interpretation
1.0	0.50 ± 0.09	0.78	0.35	Uniform fibers, stable process
1.5	0.52 ± 0.07	0.77	0.36	Acceptable, slight diameter increase
2.0	0.55 ± 0.10	0.74	0.23	Secondary jets, reduced homogeneity

The enlargement of fiber diameter after PEG incorporation may be attributed to changes in solution viscosity and electrical conductivity. PEG molecules can interact with PLA and chitosan chains through hydrogen bonding and physical entanglement, thereby increasing the effective viscosity of the spinning solution. In addition, PEG may modify the electrical conductivity of the system, which can alter the electrostatic stretching behavior of the polymer jet. Similar trends have been reported for PEG-modified electrospun membranes, where moderate PEG content improved fiber uniformity while excessive PEG caused instability in the spinning process.

Taken together, the systematic investigation identified the following formulation and processing conditions as suitable for preparing drug-loaded nanofiber membranes: PLA 10 wt%, CS 0.5 wt%, PEG 1.0 wt%, voltage 20 kV, tip-to-collector distance 15 cm, and feed rate 1.0 mL h^-1^. The selected electrospinning conditions are listed in Table 6.

**TABLE 6:** Selected electrospinning parameters for EPMC-loaded nanofibers.

Parameter	Selected condition
PLA concentration	10 wt.%
Chitosan concentration	0.5 wt.%
PEG concentration	1.0 wt.%
Applied voltage	20 kV
Tip-to-collector distance	15 cm
Feed rate	1.0 mL h^-1^

### Morphology and structure

**FIGURE 1:**
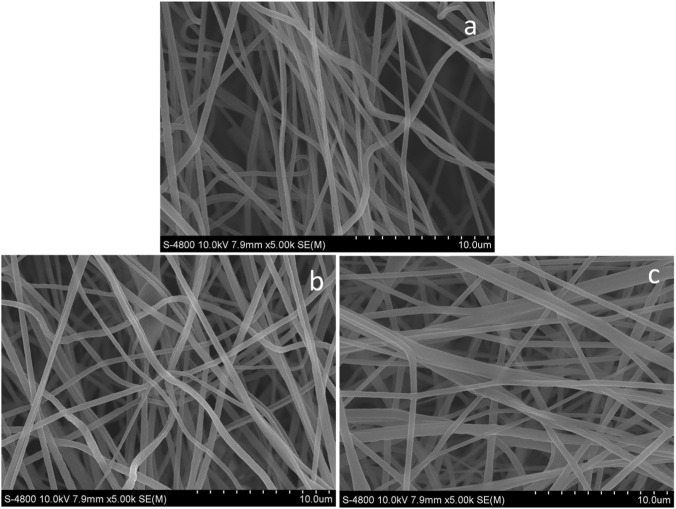
SEM images of electrospun PLA/CS/PEG nanofibers containing different EPMC loadings: **(a)** 10 wt%, **(b)** 15 wt%, and **(c)** 20 wt% EPMC.

After establishing the suitable electrospinning conditions, EPMC was incorporated into the PLA/CS/PEG system at three loading levels: 10, 15, and 20 wt% relative to polymer content. SEM images showed that all three formulations formed continuous nanofiber/microfiber networks without gross bead formation or complete jet collapse, indicating that the selected electrospinning conditions remained appropriate after drug incorporation (Figure 1). This is an important observation because plant-derived or aromatic compounds can sometimes destabilize polymer jets through changes in viscosity, polarity, or intermolecular interactions. In the present system, however, EPMC could be successfully incorporated while preserving the fibrous architecture.

The preservation of a continuous fibrous structure after drug incorporation suggests that the selected electrospinning parameters and polymer composition provided sufficient viscoelastic stability to maintain a stable polymer jet during electrospinning. In many electrospun drug-loaded systems, the addition of low-molecular-weight compounds may disrupt polymer chain entanglement or alter solution conductivity, which can result in bead formation or irregular fibers. However, the absence of bead defects in the present study indicates that the PLA/CS/PEG matrix was capable of accommodating the incorporated EPMC molecules without significantly disturbing the electrospinning process. Similar observations have been reported for other electrospun drug-loaded polymer systems where appropriate polymer concentration and solvent selection ensured stable fiber formation even after drug incorporation (Greiner and Wendorff, 2007; Bhardwaj and Kundu, 2010).

The most evident morphological effect of EPMC loading was the progressive increase in fiber diameter. At 10 wt% EPMC, the mean fiber diameter was 0.33 µm and the fibers appeared relatively fine and uniform. Increasing the EPMC content to 15 wt% led to a moderate increase in mean diameter to 0.37 µm, while 20 wt% EPMC produced the thickest fibers, with a mean diameter of 0.41 µm. This trend suggests that the addition of EPMC increased the effective solid content of the spinning solution and strengthened intermolecular interactions within the composite matrix, thereby reducing jet elongation under the electric field. The increase in fiber diameter with drug loading is consistent with the common behavior of electrospun systems carrying poorly water-soluble natural compounds, where higher loading levels tend to increase viscosity more strongly than conductivity, as summarized in Table 7.

**TABLE 7:** Effect of EPMC loading on fiber diameter.

EPMC concentration (wt.%)	Mean diameter (µm)	Maximum diameter (µm)	Minimum diameter (µm)	Morphological description
10	0.33 ± 0.12	0.53	0.20	Fine, uniform fibers
15	0.37 ± 0.11	0.56	0.22	Continuous, slightly thicker fibers
20	0.41 ± 0.11	0.70	0.25	Thickest fibers, broader size distribution

From a physicochemical perspective, the increase in fiber diameter with higher EPMC loading may also be associated with changes in the rheological properties of the spinning solution. The presence of aromatic cinnamate molecules may enhance intermolecular interactions with polymer chains through hydrophobic interactions and dipole interactions with ester groups in PLA and ether groups in PEG. These interactions can increase solution viscosity and reduce the extent of electrostatic stretching of the jet, resulting in thicker fibers. Previous electrospinning studies have reported similar effects, where increasing drug loading led to gradual enlargement of fiber diameter due to increased solution viscosity and reduced jet elongation (Li and Xia, 2004; Huang et al., 2003).

In addition, the broader size distribution observed at higher drug loading levels suggests that excessive EPMC concentration may partially disturb the homogeneity of the polymer solution, leading to localized differences in jet stretching behavior during electrospinning.

Another possible explanation for the broader diameter distribution at 20 wt% loading is the partial phase separation of drug molecules within the spinning solution. When the drug concentration approaches its solubility limit in the polymer solution, microdomains enriched with drug molecules may form, which can lead to local variations in viscosity and conductivity during electrospinning. These variations may cause fluctuations in jet stability and ultimately result in fibers with a wider diameter distribution. Similar phenomena have been reported in electrospun systems containing poorly soluble drugs or natural compounds at high loading levels (Forouzande et al., 2025; Bhardwaj and Kundu, 2010).

**FIGURE 2:**
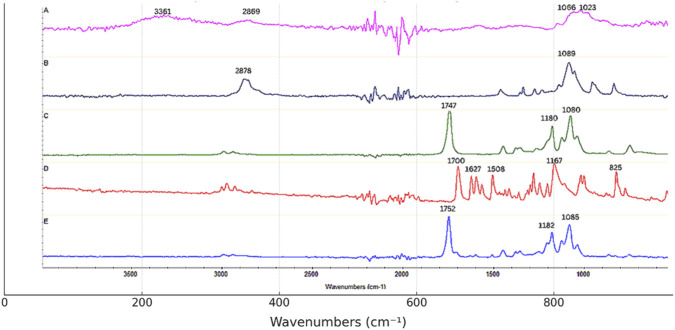
FTIR spectra of **(A)** chitosan, **(B)** PEG **(C)** PLA, **(D)** EPMC, and **(E)** electrospun PLA/CS/PEG/EPMC nanofiber membrane.

FTIR analysis further confirmed the successful incorporation of EPMC into the composite membrane (Figure 2). The spectra of individual components showed the expected characteristic bands: chitosan displayed a broad O–H/N–H stretching band around 3361 cm^-1^ and pyranose-associated bands at 1023–1066 cm^-1^; PEG showed characteristic C–H and C–O–C vibrations at 2878 and 1089 cm^-1^, respectively; PLA exhibited a strong ester carbonyl band at 1747 cm^-1^, together with C–H and C–O bands at 2994, 2944, and 1080 cm^-1^; and EPMC presented distinct aromatic and ester-related peaks, including the ester carbonyl region and the para-substituted aromatic signal around 825 cm^-1^ (Table 8).

**TABLE 8:** Main FTIR absorption bands of raw materials and composite nanofiber membrane.

Material	Main absorption band (cm^-1^)	Assignment
CS	3361	O–H / N–H stretching
CS	1023, 1066	Pyranose ring vibrations
PEG	2878	C–H stretching
PEG	1089	C–O–C stretching
PLA	1747	Ester C=O stretching
PLA	2994, 2944	C–H stretching
PLA	1080	C–O vibration
EPMC	1700	Ester C=O stretching
EPMC	1627–1508	Aromatic C=C stretching
EPMC	1167	Ester C–O vibration
EPMC	825	Para-substituted aromatic ring
Composite fiber	1752, 1085	Shifted C=O and C–O bands, indicating intermolecular interactions

In the composite nanofiber membrane, most of these characteristic absorption bands were preserved, indicating the coexistence of all four constituents in the electrospun structure. Small shifts in absorption positions, especially in the carbonyl and C–O regions, suggested the presence of molecular interactions among the components, most plausibly hydrogen bonding and dipole–dipole interactions between hydroxyl, amino, and ester-containing groups, as was shown in Table 8.

The slight shift observed in the carbonyl stretching region (around 1750 cm^-1^) in the composite nanofiber spectrum may indicate intermolecular interactions between the ester groups of PLA and the hydroxyl or amino groups of chitosan. In addition, interactions between PEG ether groups and the aromatic ester groups of EPMC may further contribute to the stabilization of the composite polymer matrix. Such intermolecular interactions are commonly observed in electrospun composite nanofibers and are often associated with improved compatibility between components and enhanced stability of drug-loaded systems (Dash et al., 2011; Rostami et al., 2024).

These interactions are relevant not only for structural integrity but also for subsequent drug release behavior, because stronger matrix–drug interactions may delay diffusion of EPMC from the fiber interior.

Furthermore, the presence of intermolecular interactions between the polymer matrix and the incorporated drug may contribute to the controlled release behavior observed in later sections of this study. Stronger interactions between polymer chains and drug molecules may retard drug diffusion from the fiber core, thereby prolonging release duration and reducing the burst release effect commonly observed in electrospun drug delivery systems.

### Surface properties

**FIGURE 3:**
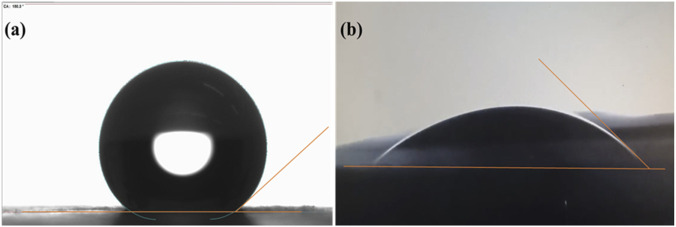
Surface wettability of electrospun membranes: **(a)** pure PLA nanofiber membrane and **(b)** PLA/PEG nanofiber membrane.

The surface properties of the membranes changed markedly after modification of the PLA matrix with PEG and EPMC. Pure PLA nanofibers exhibited a high static water contact angle of 150.3°, confirming the strongly hydrophobic character of the polymer surface (Table 9; Figure 3A). This behavior is consistent with the relatively nonpolar nature of PLA and the absence of sufficient hydrophilic surface groups capable of promoting rapid wetting. Such high hydrophobicity is undesirable for many biomedical applications because it may limit water penetration, retard initial drug diffusion, and reduce interaction with biological fluids.

**TABLE 9:** Surface wettability of representative nanofiber membranes.

Sample	Contact angle	Surface characteristic
PLA	150.3°	Strongly hydrophobic
PLA/PEG	Not measurable (instant penetration)	Strongly hydrophilic
PLA/PEG/EPMC	Not measurable (instant penetration)	Strongly hydrophilic

The hydrophobic nature of PLA nanofibers has been widely reported in electrospinning studies, where contact angles typically exceed 120° due to the combined effect of polymer chemistry and nanoscale surface roughness. The ester backbone of PLA lacks strongly hydrophilic functional groups, and the fibrous microstructure can further amplify hydrophobic behavior through the Cassie–Baxter effect. As a result, pristine PLA electrospun membranes often require surface modification or polymer blending to improve wettability for biomedical applications such as tissue engineering or drug delivery (Li and Xia, 2004; Bhardwaj and Kundu, 2010).

By contrast, incorporation of PEG markedly increased the surface hydrophilicity of the membrane (Figure 3B). When PEG was incorporated into the PLA-based system, the deposited water droplet rapidly spread and completely penetrated the membrane, making conventional static contact angle measurement impractical. The same phenomenon was observed for the EPMC-containing membrane. These results indicate a major transition from hydrophobic to strongly hydrophilic surface behavior, as summarized in Table 9. Since the intended application of the material involves contact with PBS during drug release, wettability behavior under physiological buffer conditions is also relevant and warrants further investigation in future studies.

PEG likely contributed to this effect through its hydrophilic ether groups and its tendency to enrich the fiber surface during electrospinning (Samokhin et al., 2023). The addition of EPMC may also have enhanced water uptake by altering fiber microstructure and by contributing polar functional groups to the system. PEG is well known for its strong hydrophilic character and high affinity for water molecules due to the presence of repeating ether groups (–C–O–C–) along the polymer chain. During electrospinning, PEG molecules may preferentially migrate toward the fiber surface because of differences in surface energy between PEG and PLA. This surface enrichment can significantly increase membrane wettability and facilitate rapid water absorption. Similar improvements in hydrophilicity have been reported in PLA/PEG electrospun systems, where PEG incorporation dramatically reduced contact angles and enhanced water permeability of the nanofiber membranes (Dash et al., 2011; Huang et al., 2003).

In addition, the incorporation of EPMC may further modify surface wetting behavior. Although EPMC is not strongly hydrophilic, its aromatic ester structure can interact with PEG and PLA through dipole interactions, potentially altering the surface microstructure and increasing water accessibility within the fibrous network. The combined presence of PEG and EPMC may therefore create microdomains with enhanced water affinity, contributing to the rapid penetration of water droplets observed experimentally.

**FIGURE 4:**
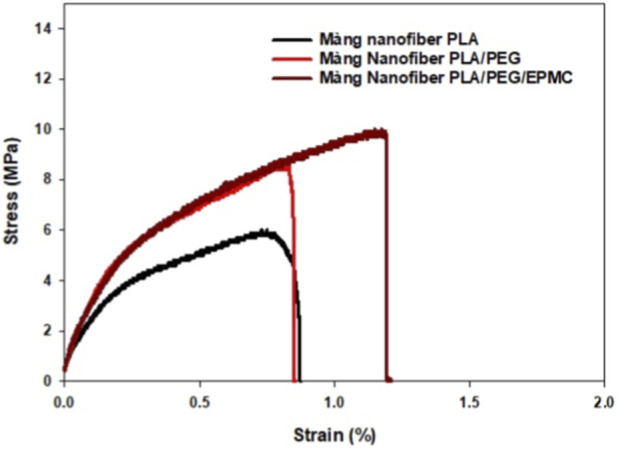
Representative tensile stress–strain curves of electrospun nanofiber membranes: PLA, PLA/PEG, and PLA/PEG/EPMC.

Mechanical testing showed that modification of PLA with PEG and EPMC reduced tensile strength (Figure 4; Table 10). Pure PLA exhibited the highest tensile stress, approximately 10.2 MPa. After addition of PEG, the value decreased to approximately 9.2 MPa, and with further incorporation of EPMC the tensile stress dropped more clearly to about 6.3 MPa, as shown in Table 10. This reduction indicates that PEG and EPMC, while favorable for hydrophilicity and drug transport, partially weakened the dense intermolecular packing of the PLA network.

**TABLE 10:** Tensile stress of representative fiber membranes.

Sample	Maximum tensile stress (MPa)	Interpretation
PLA	∼10.2	Highest mechanical integrity
PLA/PEG	∼9.2	Slight reduction due to plasticizing effect
PLA/PEG/EPMC	∼6.3	Lower strength, higher flexibility and hydrophilicity

The reduction in tensile strength after PEG incorporation can be attributed to the plasticizing effect of PEG molecules within the PLA matrix. PEG chains may increase the mobility of polymer segments and reduce intermolecular interactions between PLA chains, leading to decreased stiffness and lower tensile strength. This plasticizing behavior has been widely reported in PLA-based composite systems where PEG is introduced to improve flexibility and processability (Auras et al., 2004).

Furthermore, the additional decrease in tensile strength observed after EPMC incorporation may result from the presence of small-molecule additives within the polymer network. Drug molecules embedded within electrospun fibers may act as structural discontinuities that disrupt polymer chain packing and reduce mechanical cohesion. Similar mechanical weakening has been observed in many electrospun drug-loaded systems, where increased drug loading levels often lead to reduced tensile strength due to decreased intermolecular polymer interactions (Greiner and Wendorff, 2007).

In practical terms, this suggests a trade-off between mechanical integrity and functional performance. For drug-delivery membranes intended for flexible biomedical use rather than load-bearing applications, such a reduction may still be acceptable, provided that the membrane maintains sufficient structural coherence during handling and immersion.

Indeed, moderate reduction in mechanical strength is often acceptable or even beneficial for biomedical membranes, particularly when improved flexibility and hydrophilicity enhance biological compatibility and drug release performance. The balance between mechanical stability and functional properties is therefore an important consideration when designing electrospun polymer systems for drug delivery applications.

### Drug loading efficiency

The incorporation efficiency of EPMC within the electrospun membrane decreased as the theoretical drug loading increased. The 10 wt% formulation showed the highest encapsulation efficiency, reaching 35.08% ± 3.68%. When the EPMC content was increased to 15 wt%, the efficiency declined to 24.06% ± 2.96%, and further decreased to 18.03% ± 1.16% at 20 wt%. This pattern suggests that drug retention in the nanofiber system was limited by the compatibility and solubilization capacity of the polymer matrix, as summarized in Table 11.

**TABLE 11:** Encapsulation efficiency of EPMC in PLA/CS/PEG nanofiber membranes.

Initial EPMC loading (% wdrug/wpolymer)	Encapsulation efficiency (%)
10	35.08 ± 3.68
15	24.06 ± 2.96
20	18.03 ± 1.16

A similar trend has frequently been reported in electrospun drug-loaded nanofiber systems, where increasing the theoretical drug loading does not necessarily lead to higher encapsulation efficiency. Instead, excessive drug concentration may exceed the solubilization capacity of the polymer solution, resulting in partial phase separation or incomplete incorporation of drug molecules within the polymer matrix. In such cases, a portion of the drug may remain near the fiber surface or become lost during electrospinning due to solvent evaporation and jet stretching processes (Bhardwaj and Kundu, 2010).

A plausible explanation lies in the physicochemical nature of EPMC. Although it is soluble in the organic phase used for spinning, its increasing concentration may approach the saturation threshold of the polymer solution. At high loading levels, part of the drug may segregate, crystallize locally, or become poorly entrapped during jet stretching and solvent evaporation. In addition, stronger intermolecular association between EPMC and the polymer matrix may hinder complete extraction during the encapsulation assay, contributing to the apparent decline in measured efficiency.

From a molecular perspective, the incorporation of EPMC into the PLA/CS/PEG nanofiber matrix may involve multiple types of interactions, including hydrophobic interactions between the aromatic ring of EPMC and the hydrophobic backbone of PLA, as well as hydrogen bonding interactions with chitosan and PEG. At moderate concentrations, these interactions may facilitate drug entrapment within the polymer matrix. However, when the drug loading becomes excessive, drug–drug interactions may become dominant, leading to aggregation or microcrystalline domains within the polymer solution. Such aggregation can reduce the effective entrapment of drug molecules during electrospinning and ultimately lower encapsulation efficiency (Greiner and Wendorff, 2007).

Another factor that may contribute to the reduced encapsulation efficiency at higher loading levels is the dynamic nature of the electrospinning process. During jet formation and elongation, rapid solvent evaporation and strong electrostatic forces may cause migration of small molecules toward the fiber surface. As a result, a portion of the drug may be lost during fiber formation or become weakly associated with the fiber surface rather than being fully entrapped within the polymer matrix. This phenomenon has been observed in several electrospun drug delivery systems, particularly when low-molecular-weight compounds are incorporated into polymer nanofibers (Huang et al., 2003).

From an application perspective, the 10 wt% sample exhibited the highest retention efficiency, but the absolute amount of drug incorporated per unit membrane mass was lower than in the 15 wt% sample. Therefore, the 15 wt% formulation represented a more balanced condition, combining acceptable encapsulation efficiency with a higher amount of available active compound.

This observation highlights an important consideration in the design of electrospun drug delivery systems, where a suitable balance must be achieved between encapsulation efficiency and drug loading capacity. While lower drug loading may lead to higher encapsulation efficiency, it may not provide sufficient therapeutic dosage. Conversely, excessively high loading may compromise drug distribution within the fibers and reduce overall encapsulation efficiency. In many electrospun drug delivery systems, intermediate loading levels have been found to provide the most favorable compromise between efficient drug incorporation and adequate drug content for sustained release applications (Li and Xia, 2004).

### Drug release behavior

**FIGURE 5:**
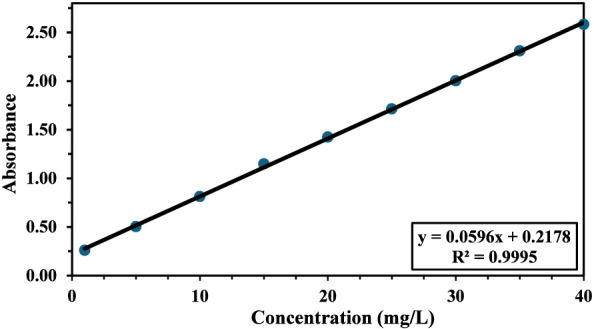
Calibration curve of EPMC in PBS buffer (pH 7.4) used for quantitative determination of drug loading and release.

The *in vitro* release behavior of EPMC was evaluated in PBS at pH 7.4. Before quantification, a calibration curve was established by UV–Vis spectroscopy. The maximum absorption wavelength of EPMC in the selected medium was identified at 306.2 nm, and the calibration curve exhibited excellent linearity, with a coefficient of determination of 0.9995 (Figure 5). This confirmed the suitability of the analytical method for quantifying EPMC release from the membranes.

The high linearity of the calibration curve indicates that the UV–Vis spectroscopic method is reliable for quantifying EPMC concentration in the release medium. Accurate calibration is particularly important in drug release studies involving electrospun nanofibers because the released drug concentration may vary widely during the release period. A well-defined calibration curve ensures that cumulative release data accurately reflect the amount of drug diffusing from the polymer matrix into the surrounding medium.

The release profiles showed distinct differences among the three formulations. The 10 wt% and 15 wt% samples both released approximately half of their encapsulated drug after 24 h, whereas the 20 wt% sample released only 42.85% ± 6.85% over the same period. This indicates that excessive EPMC loading did not enhance release performance; instead, it may have promoted stronger retention within the matrix and reduced effective diffusion.

In electrospun drug delivery systems, the drug release rate is often governed by several factors, including fiber diameter, drug distribution within the fibers, polymer–drug interactions, and the hydrophilicity of the polymer matrix. When the drug loading becomes excessively high, drug molecules may form aggregates or microdomains within the polymer structure, which can limit the diffusion of drug molecules from the fiber core to the surrounding medium. Such aggregation may also strengthen drug–drug interactions, thereby slowing the release process (Bhardwaj and Kundu, 2010).

The 10 wt% sample displayed very limited release during the first hour (0.90% ± 0.64%), followed by a marked increase from the second hour onward. The 15 wt% formulation also exhibited low initial release, but the subsequent release was steadier and reached the highest cumulative value at 24 h (51.32% ± 7.11%). In contrast, the 20 wt% sample showed a relatively high initial release at 1 h (18.59% ± 2.75%), followed by slower subsequent release, suggesting an uneven drug distribution, with a larger fraction of the drug located near or at the fiber surface. This behavior is less desirable for controlled release applications because it combines an initial burst with incomplete later release, as shown in Table 12.

**TABLE 12:** Cumulative release of EPMC from nanofiber membranes in PBS pH 7.4.

Time (h)	10% loading	15% loading	20% loading
1	0.90 ± 0.64	2.02 ± 2.47	18.59 ± 2.75
2	17.71 ± 3.08	5.57 ± 5.96	22.34 ± 1.01
3	28.95 ± 5.16	10.14 ± 6.75	26.07 ± 0.91
4	37.15 ± 8.47	13.29 ± 6.94	28.20 ± 1.61
5	40.62 ± 5.17	17.37 ± 6.88	29.79 ± 2.17
6	41.14 ± 5.26	24.33 ± 9.71	31.08 ± 2.93
7	44.06 ± 5.08	25.47 ± 7.95	32.32 ± 3.26
8	46.03 ± 7.15	25.84 ± 7.78	33.91 ± 3.62
24	50.30 ± 6.76	51.32 ± 7.11	42.85 ± 6.85

The burst release observed for the 20 wt% sample during the first hour is a common phenomenon in electrospun drug-loaded systems. It typically occurs when a portion of the drug is located near the fiber surface or weakly associated with the polymer matrix. Upon contact with the release medium, these surface-associated drug molecules rapidly diffuse into the surrounding solution, producing an initial burst effect. Several studies have reported similar burst release behavior in electrospun nanofibers containing small-molecule drugs or natural compounds (Greiner and Wendorff, 2007).

By contrast, the lower initial release observed in the 10 wt% and 15 wt% formulations suggests that most of the EPMC molecules were successfully embedded within the interior of the fibers rather than on the surface. As water penetrates the hydrophilic PLA/CS/PEG matrix, drug molecules gradually diffuse through the polymer network, resulting in a more controlled release pattern. The presence of PEG and chitosan likely enhanced water uptake and facilitated diffusion pathways within the nanofiber structure, thereby promoting sustained drug release over time (Huang et al., 2003).

The release data suggest that the 15 wt% EPMC membrane offered the best balance between low initial loss, gradual diffusion, and substantial cumulative release after 24 h. This pattern is desirable for biomedical delivery materials that require sustained exposure rather than rapid depletion of the active compound.

The relatively steady release profile observed for the 15 wt% formulation may reflect a suitable balance between drug loading and polymer matrix structure. At this loading level, sufficient drug molecules are incorporated to achieve meaningful release levels, while polymer–drug interactions remain moderate enough to allow gradual diffusion through the fiber network. Consequently, this formulation appears to provide the most favorable release characteristics for controlled drug delivery applications.

#### Release kinetics

To better understand the release mechanism, the experimental release data were fitted to zero-order, first-order, Higuchi, and Korsmeyer–Peppas models. Among the investigated models, the Korsmeyer–Peppas equation showed the best fit for all three formulations, as indicated by the highest correlation coefficients in each case. This finding demonstrates that EPMC release from the composite nanofiber membranes cannot be explained by simple concentration-dependent dissolution alone, but rather by a more complex transport process involving both diffusion and matrix-related effects.

Mathematical modeling of drug release kinetics is widely used to elucidate the mechanisms governing drug transport from polymer-based delivery systems. Among the commonly applied models, the Korsmeyer–Peppas equation is particularly useful for describing drug release from polymeric matrices when more than one transport mechanism is involved. This empirical model accounts for the combined effects of diffusion, polymer swelling, and structural relaxation, which are frequently observed in electrospun nanofiber systems (Peppas and Sahlin).

For the 10 wt% and 20 wt% formulations, the release exponent n was lower than 0.5, indicating quasi-Fickian behavior. In these samples, drug transport was governed predominantly by diffusion through the polymer matrix, with only limited contribution from matrix relaxation. The 15 wt% sample, however, exhibited an n value of 0.70, corresponding to a non-Fickian or anomalous release mechanism. This suggests that drug release from this formulation was controlled by a combination of diffusion and polymer-chain relaxation or structural rearrangement, as summarized in Table 13.

**TABLE 13:** Kinetic parameters of EPMC release from PLA/CS/PEG nanofiber membranes.

Kinetic model	Parameter	10% loading	15% loading	20% loading
Zero-order	R^2^	0.6756	0.9558	0.7476
	K0	3.34	2.50	2.71
First-order	R^2^	0.4386	0.7390	0.8314
	K1	0.28	0.25	0.27
Higuchi	R^2^	0.8617	0.9748	0.9305
	KH	13.92	9.07	11.43
Korsmeyer–Peppas	R^2^	0.8924	0.9844	0.9983
	KKP	19.03	5.63	19.52
	n	0.36	0.70	0.25

In polymeric drug delivery systems, the value of the release exponent n provides insight into the dominant release mechanism. For cylindrical or fibrous matrices, an n value below 0.5 typically indicates Fickian diffusion, where drug molecules migrate through the polymer network primarily due to concentration gradients. Values between approximately 0.5 and 1.0 correspond to anomalous or non-Fickian transport, reflecting the combined influence of molecular diffusion and polymer relaxation processes. Such behavior often arises in polymer matrices that undergo swelling, partial structural rearrangement, or changes in chain mobility during drug release (Ritger and Peppas, 1987).

The kinetic analysis also revealed that the Higuchi model produced relatively high correlation coefficients, particularly for the 15 wt% formulation (*R* ^2^ = 0.9748). This indicates that diffusion from a porous matrix contributed significantly to the release process. However, the slightly higher correlation obtained with the Korsmeyer–Peppas model suggests that diffusion alone does not fully describe the release mechanism in this system.

The applicability of the Higuchi model suggests that drug diffusion from the porous nanofiber network played an important role in the release process. Electrospun membranes possess a highly porous structure with large surface area and interconnected diffusion pathways, which facilitate solvent penetration and drug migration. Nevertheless, because the PLA/CS/PEG matrix may also undergo hydration and partial polymer relaxation, the release behavior cannot be described solely by diffusion. The improved fit of the Korsmeyer–Peppas model therefore reflects the combined contribution of diffusion and matrix relaxation processes (Higuchi, 1963).

Although the 20 wt% sample achieved the highest *R* ^2^ value for the Korsmeyer–Peppas model (0.9983), its n value was relatively low (0.25), indicating predominantly diffusion-controlled transport. This observation is consistent with the burst release behavior observed in the early release stage, where drug molecules located near the fiber surface rapidly diffuse into the surrounding medium.

The relatively low n value observed for the 20 wt% formulation may also reflect heterogeneous drug distribution within the fibers. At high drug loading levels, a portion of the drug may accumulate near the fiber surface during electrospinning due to solvent evaporation and phase separation phenomena. Such surface-localized drug molecules can diffuse rapidly into the release medium, producing a diffusion-dominated release mechanism with limited contribution from polymer relaxation (Greiner and Wendorff, 2007).

By contrast, the 15 wt% formulation exhibited a more balanced release behavior. Its non-Fickian transport mechanism suggests that drug diffusion occurred concurrently with structural relaxation of the polymer matrix, which is consistent with the sustained release profile observed in the previous section.

The anomalous transport behavior observed for the 15 wt% formulation may result from a favorable balance between drug loading, polymer composition, and fiber morphology. At this loading level, drug molecules are likely distributed more uniformly throughout the nanofiber network, allowing diffusion to occur gradually while polymer chains simultaneously undergo hydration and relaxation in the aqueous medium. Such coupled diffusion–relaxation mechanisms are often associated with more controlled and sustained release performance in electrospun drug delivery systems.

Despite the promising results obtained in this study, several limitations should be acknowledged. The drug release experiments were conducted only under *in vitro* conditions, and the behavior of the nanofiber membranes in complex biological environments remains to be investigated. Furthermore, long-term stability and biodegradation of the PLA/CS/PEG system were not evaluated and should be addressed in future studies.

## Conclusion

Electrospun PLA/chitosan/PEG nanofiber membranes loaded with ethyl *p*-methoxycinnamate (EPMC) were successfully fabricated and evaluated as a biodegradable drug delivery system. Suitable formulation composition and electrospinning conditions were identified for producing uniform and stable nanofibers, including 10 wt% PLA, 0.5 wt% chitosan, 1.0 wt% PEG, an applied voltage of 20 kV, a tip-to-collector distance of 15 cm, and a feed rate of 1.0 mL h^-1^. Incorporation of EPMC increased fiber diameter and significantly enhanced surface wettability, while moderately reducing mechanical strength due to the plasticizing effects of PEG and the presence of drug molecules within the polymer matrix. Encapsulation efficiency decreased as theoretical drug loading increased, indicating a limited solubilization capacity of the polymer system for EPMC. *In vitro* release studies demonstrated sustained drug release over 24 h, and kinetic analysis showed that the release behavior followed the Korsmeyer–Peppas model, suggesting a combined diffusion–relaxation transport mechanism. Among the tested formulations, the membrane containing 15 wt% EPMC exhibited the most balanced performance in terms of drug incorporation and release behavior, indicating that PLA/chitosan/PEG electrospun nanofibers represent a promising platform for the controlled delivery of bioactive natural compounds.

## Data Availability

The raw data supporting the conclusions of this article will be made available by the authors, without undue reservation.
